# Fabrication and Characterization of a Composite Ni-SDC Fuel Cell Cathode Reinforced by Ni Foam

**DOI:** 10.3390/ma15144891

**Published:** 2022-07-14

**Authors:** Gabriela Komorowska, Tomasz Wejrzanowski, Jan Jamroz, Agnieszka Jastrzębska, Wojciech Wróbel, Shu-Yi Tsai, Kuan-Zong Fung

**Affiliations:** 1 Faculty of Materials Science and Engineering, Warsaw University of Technology, Woloska 141, 02-507 Warsaw, Poland; agnieszka.jastrzebska@pw.edu.pl; 2 Faculty of Physics, Warsaw University of Technology, Koszykowa 75, 00-662 Warsaw, Poland; jan.jamroz.dokt@pw.edu.pl (J.J.); wojciech.wrobel@pw.edu.pl (W.W.); 3 Hierarchical Green-Energy Materials (Hi-GEM) Research Center, National Cheng Kung University, Tainan 70101, Taiwan; willxkimo@yahoo.com.tw (S.-Y.T.); kzfung@ncku.edu.tw (K.-Z.F.); 4 Department of Materials Science and Engineering, National Cheng Kung University, Tainan 70101, Taiwan

**Keywords:** cerium oxide, fuel cell, cathode, porosity, tape casting, nickel, foam

## Abstract

High-temperature fuel cells (namely, molten carbonate and solid oxide; MCFCs and SOFCs) require the cathode to be designed to maximize oxygen catalytic reduction, oxygen ion transport, electrical conductivity, and gas transport. This then leads to the optimization of the volume fraction and morphology of phases, as they are a pathway for electrons, ions, and gases to be continuous and self-interpenetrating. Apart from the functional properties, the cathode must be mechanically stable to prevent cracking during fuel cell assembly and operation. The manufacturing process of the composite cathode was optimized to meet such requirements in this research work. The tape casting technique and further firing process were used to fabricate the cathodes. The slurry for the green tape was composed of nickel (Ni), cerium oxide doped with samarium oxide (SDC), water (solvent), and an organic binder (which becomes pore space after firing). Each of these elements is necessary for the effective transport of specific species: electrons, oxygen, ions, and gas particles, respectively. Moreover, the nickel foam was embedded into the powder-based structure to improve mechanical strength. The study involved many technological issues, such as the effect of the SDC fraction on the cathode microstructure, mechanical strength, and chemical stability at high temperatures, and also involved environmental issues.

## 1. Introduction

Fuel cells are known as highly efficient and environmentally friendly power generation devices. High-temperature fuel cells operate at temperatures above 500 °C. Due to the higher working temperatures, they can achieve higher reaction rates with cheaper catalysts in comparison with low-temperature fuel cells [1]. In addition, they can be supplied with different fuels such as natural gas or methane and are not sensitive to contamination [2]. On the other hand, they are more complex than low-temperature fuel cells. They need extra devices such as pre-heaters, a cooling system, and complex software, and they are more expensive to manufacture. High-temperature fuel cells work well and are more productive in stable, stationary applications [1]; thus, they are an adequate technology for distributed energy production systems [3].

Two types of cells operating at higher temperatures are distinguished: molten carbonate fuel cells (MCFCs) and solid oxide fuel cells (SOFCs). The first uses a eutectic carbonate mixture as an electrolyte, which conducts CO_3_^2−^ ions at the operating temperature (650 °C). The matrix, which is in the form of a porous, non-conductive ceramic (LiAlO_2_) film that keeps the fluid inside the pores by capillary forces, protects the cell against electrolyte leakage. Both electrodes are made of nickel, which has good catalytic properties at the operating temperature. The cathode is oxidized in situ to NiO during the initial operation of the fuel cell. Usually, the electrodes and matrix are manufactured by tape casting. This method allows for the production of large-size, two-dimensional, thin, porous tapes from powder suspensions [4].

In solid oxide fuel cells, the anode is usually made of nickel, and the cathode is made of perovskites such as LSM (La_0.8_Sr_0.2_MnO_3_) or LSCF (La_0.6_Sr_0.4_Co_0.2_Fe_0.8_O_3_) [5]. The electrolyte is an oxide, ion conductive, non-porous, ceramic layer. One of the most popular electrolyte materials is doped oxides such as yttria-stabilized zirconia (YSZ), gadolinia-doped ceria (GDC), and samarium-doped ceria (SDC) [6]. Ceria-based electrolytes achieve higher ionic conductivity, which allows for a lower working temperature (IT-SOFC) [7,8,9,10]. At the same time, its drawback is a high level of electronic conductivity, which can lead to lower fuel cell power density. On the other hand, this type of property, called mixed ionic-electronic conductivity (MIEC), is of interest because it can increase the reaction rate of fuel gases at the triple-phase boundary [11]. Typical manufacturing methods for all three components are techniques such as tape casting, screen printing, pressing, spraying, and coating, which lead to the thickness of layers being within the range of 2–500 μm [12,13]. Layer deposition techniques are also applied when thin films are required (up to ~100 nm) [14].

The reaction rate on the cathode side is the limiting factor for SOFC and MCFC performance [15]. This rate depends on chemical composition [16,17,18] and microstructure [19,20]. In traditional SOFC technology, the main problems are the high working temperatures, which creates a complicated and expensive manufacturing process for the solid ceramic electrolyte [21]. When the solutions characteristic of both technologies are combined, it is possible to eliminate many disadvantages of the individual systems and improve efficiency and durability.

Recently, such combined systems have been extensively studied, but those studies are unsystematic and significantly differ from each other in the type of ceramics used as oxide ion electrolytes, the method of production, the operating parameters, and therefore, the achieved efficiencies. The reported maximum power densities vary from 224 mWcm^−2^ [22] to 1704 mWcm^−2^ [23] at 650 °C, whereas the reference value for a conventional MCFC is 137 mWcm^−^^2^ [24]. Depending on whether the author had previously dealt with SOFCs or MCFCs, they are named differently: electrolyte combined in MCFCs [24], composite electrolyte in IT-FCs [25], or composite electrolyte in SOFCs [22]; these are just a few of the different nomenclature variants.

This combined system consists of three hybrid elements. The electrolyte was composed of SDC (electrolyte in IT-SOFCs) and the eutectic mixture of carbonate (electrolyte in MCFCs). The anode was manufactured from nickel, which is typically used for both types of high-temperature fuel cells. The cathode was a mixture of Ni (cathode in MCFCs) and SDC. The addition of the electrolyte material to the cathode was specific to IT-SOFCs. This introduced the conduction paths of oxygen ions in the cathode surface’s vicinity, which accelerates the reaction rate at the triple-phase boundary. Additionally, it provides continuous oxygen ion conduction paths through the electrolyte towards the anode. This research aims to obtain a composite Ni-SDC cathode from an aqueous suspension by tape casting as the first step towards a systematic study of the composite system.

Water was chosen as a solvent because it is cheap and friendly to the environment and researchers, unlike the usually used organic solvents. On the other hand, obtaining a ceramic suspension with adequate powder dispersion is more challenging due to the zeta potential issue [26]. The tape casting method is a widely known technology for producing tapes with a large surface area. Its usage potentially allows for increasing the scale of production and commercializing. In SOFCs, sintering is most often used, which does not provide the same potential.

## 2. Materials and Methods

### 2.1. Materials

Powders of nickel (T255™ nickel powder, VALE) and samarium oxide-doped cerium oxide (SDC), manufactured using the solid-state reaction process by the Department of MSE, National Cheng Kung University, were used as the main elements for cathode fabrication. The starting materials to obtain SDC were CeO_2_ (99% pure) and Sm_2_O_3_ (99.99% pure). Substrates were ball-milled with zirconia balls in a high-density polypropylene bottle for one day. After drying, the powders were calcined at 1200 °C for 7 h to obtain SDC powders. Finally, each powder was sieved using a 200 mesh to obtain uniform particle sizes.

### 2.2. Slurries Preparation

A planetary centrifugal vacuum mixer, the THINKY ARV-930TWIN, was used to prepare an aqueous suspension. The process consisted of several steps. Firstly, the plasticizer (PEG400) and polymeric binders (carboxymethyl cellulose and hydroxypropyl methylcellulose) were mixed at 200 rpm for 5 min under vacuum conditions (40 kPa). Secondly, the solvent (distilled water), additional plasticizer (glycerin), defoamer (AGITAN 282), and dispersant (METOLAT 388) were added and homogenized at 600 rpm for 10 min under vacuum conditions (40 kPa). Thirdly, to obtain a complex porous structure that was previously optimized [20], two porogens (starch and polyvinyl butyral; Mowital B 60 H, Kuraray) were added and mixed at 800 rpm for 10 min under vacuum conditions (0.6 kPa). Finally, the nickel and SDC powders were added as different volume ratios of SDC (20%, 40%, 50%, and 60%). The slurries were again homogenized at 800 rpm for 45 min under vacuum conditions (0.6 kPa).

### 2.3. Cathode Preparation

**Figure 1:**
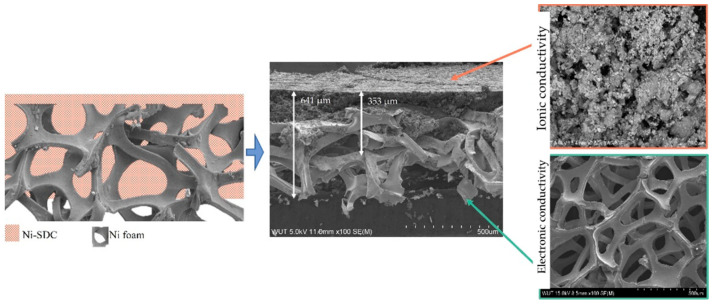
Construction of a two-layer Ni-SDC cathode.

The two-layer cathode was prepared using tape casting. The first layer was a commercial nickel foam (Gelon Lib Co., Linyi, China) with a 0.5 mm thickness, 100 ppi, 250 g/cm^2^ surface density, and 85% open porosity. The nickel foam was intended to increase the mechanical properties of the cathode [27] and add bigger pores, which are more easily penetrated by reaction gases. This solution is innovative and has a pending patent application. The second layer was tape manufactured from the previously prepared slurry (Figure 1). The nickel-SDC layer was cast onto the nickel foam with a casting speed of 2 mm/s, through a “doctor blade” gap (0.8 mm), and dried for 24 h at room temperature. Subsequently, the cathode was sintered in a reducing atmosphere of an N_2_ + 5% H_2_ mixture in a three-step process. The elastic green tape was annealed at 200 °C for two hours to remove volatile compounds, heated at 400 °C for another two hours to burn out the organic compounds, and sintered at 800 °C for one hour. A pure nickel cathode was also manufactured to compare the effect of the added SDC to the reference MCFC cathode [28]. During sintering, a reduced atmosphere was used to protect against nickel oxidation. When nickel transforms to nickel oxide, the sintering temperature increases, and it is difficult to obtain a high porous microstructure with adequate mechanical properties.

### 2.4. Characterization of the Cathodes

The microstructures of the cathodes and their cross-sections before and after the sintering process were characterized using scanning electron microscopes: the Hitachi SU8000, Hitachi SU5500, and Hitachi SU70 (in magnetic sample mode). Backscattered electron (BSE) and secondary electron (SE) detectors were used. The particle characterization of nickel powders was conducted in water using binary dynamic image analysis (Particle Insight 2.69; particle shape analyzer). The chain aspect ratio parameter was used to analyze this, which is the ratio of length chains to width chains. The SDC particle size distributions were measured using a laser-scattering particle size distribution analyzer, the HORIBA LA-950, in isopropanol using ultrasonic mode.

The critical parameters for the cathode in the fuel cell are porosity and a specific surface area. Open porosity allows access to reaction gases on the catalytic surface. The specific surface area determines the number of sites for electrocatalytic reactions to occur. The porosity of cathodes with different volume ratios of SDC were measured using the buoyancy method. This method is based on weighing a dry sample and comparing the mass when it is soaked by water and in water. Then, the apparent density and the open and closed porosity are calculated from these three masses. Surface area and porosity were evaluated by the physical nitrogen sorption method using the Quadrasorb SI (Quantachrome Instruments, Germany) equipped with the FloVac Degasser. For this purpose, about 1 g of the cathode was dried for 24 h in 250 °C. The nitrogen adsorption was carried out in a liquid nitrogen bath at −195 °C. The specific surface area was calculated using the Brunauer–Emmett–Teller method (BET). Total porosity, pore size distribution, and mean pore size parameters were evaluated using the Barrett–Joyner–Halenda (BJH) method. In particular, the advanced calculations obtained the BET surface area (S_BET_), pore surface area (S_pore_), external surface area (S_external_), micropore surface area (S_micropore_), pore volume (V_pore_), micropore volume (V_micropore_), and average pore diameter (D_pore_).

X-ray powder diffraction data were collected on a PANalytical Empyrean Series 2 diffractometer fitted with a PIXcel3D detector, using Cu Kα radiation (λ_1_ = 1.54056 Å and λ_2_ = 1.54439 Å). Data suitable for detailed Rietveld refinement were collected in the 2θ range of 5–125°, in steps of 0.0131°, with an effective scan time of 250 s per step. Calibration was carried out with an external Si standard. Structure refinement was performed with the GSAS-II suite of programs [29]. A cubic model in the space group Fm-3m was used for all refinements, both in the case of SDC and Ni phases. In the case of SDC, Ce and Sm atoms were located on the 4a site (000), whereas the O atom was located on the 8c site (0.25 0.25 0.25). One atomic site associated with Ni located on the 4a site (000) was applied for the Ni phase. The crystal and refinement parameters are given in supporting documentation.

A tensile test was performed to characterize the mechanical strength of the materials. For each test, samples of 10 mm × 5 mm × 0.6 mm were cut from the larger tape. Mechanical testing was carried out using an MTS Tytron 250 system.

## 3. Results and Discussion

### 3.1. Characterization of Substrates

**Figure 2:**
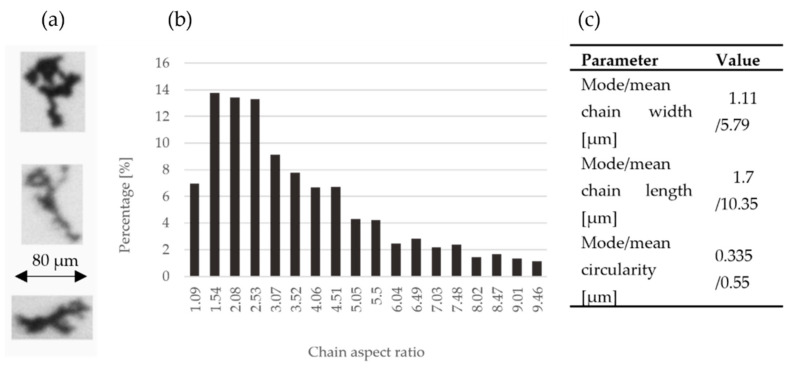
Binary dynamic image analysis for Ni powder: (**a**) images of Ni powder; (**b**) percentages of the individual values of chain aspect ratio graph; (**c**) characteristic parameters of powder.

The primary substrates of the composite cathodes were Ni and SDC powders. The first of them was in the form of chains, which created a very desirable porous microstructure after sintering (Figure 2). Binary image analysis showed that aspect ratios were different, but almost 50% of particles had an aspect ratio between 1.5 and 2.5. The mode circularity was 0.335 µm and the mean circularity 0.55 µm ± 0.178 µm, which indicates the ellipticity of the equivalent circuit.

**Figure 3:**
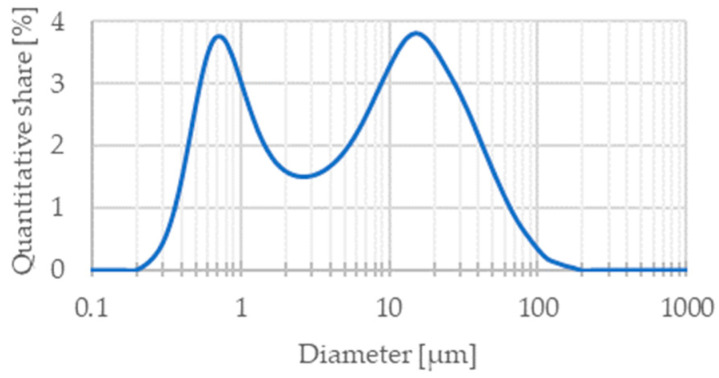
Particle size distribution of SDC powder by laser-scattering method.

**Figure 4:**
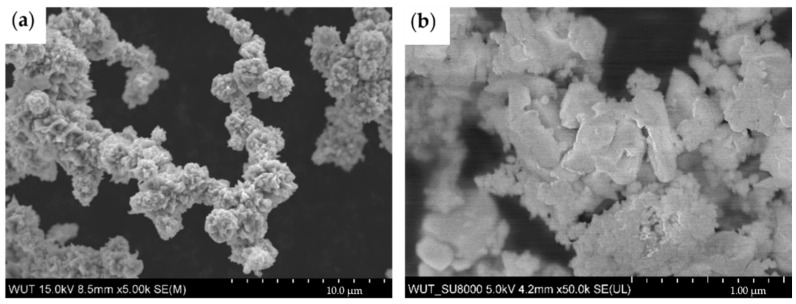
SEM images of (**a**) Ni powder, (**b**) SDC powder.

The mode chain width is an important parameter, which allows one to compare the size of Ni and SDC particles. For nickel, it is equal to 1.1 µm. The analysis of SDC powder by laser scattering showed two fractions. The first peak was about 766 nm and the second was 15 µm (Figure 3). It shows a high aggregation tendency, which was confirmed by SEM analyses (Figure 4). This implies that both powders had similar sizes, but when analyzing the SEM images of the Ni-SDC cathode after sintering, it was seen that the SDC powder is much smaller. This was caused by a significant increase in the width of nickel chains during the thermal process (two–three times) when the SDC powder remained unchanged, or during the technological process the agglomerates were broken up, and significant nano-sized fractions were formed.

### 3.2. Optimization of the Manufacturing Process of the Ni-SDC Cathode’s Aqueous Suspension

**Figure 5:**
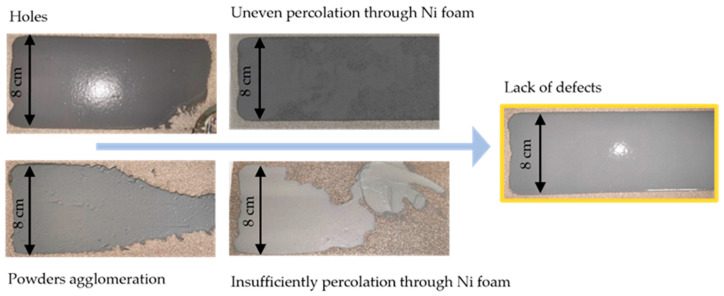
Types of defects occurring during Ni-SDC cathode optimization.

The chemical composition and manufacturing process of the reference Ni cathode was optimized previously by the T. Wejrzanowski group [30]. The addition of SDC powder caused a change in the suspension castability (insufficient percolation through Ni foam) and the appearance of defects. The defects were in the form of holes or powder agglomerations (Figure 5). The SDC-Ni cathode volume fraction of dispersant was increased by 425%. This led to the lack of agglomerates. To minimize the occurrence of holes, the type of antifoam was changed. Optimal percolation through Ni foam was achieved by a water volume fraction of 12%. For a small amount of SDC (below 20% volume fraction), the solvent volume must be the same as in the pure Ni cathode. This indicates the significant effect of the SDC powder on reducing the castability of the slurry. The manufacturing method was also optimized. The final mixing stage was extended from 15 to 45 min to combine the surface-active ingredients with the ceramic powder better, and a vacuum was applied for degassing.

The Ni-SDC cathode was optimized to have a similar thickness as the Ni cathode (Table 1) as compared to the results from the earlier studies which were considered optimal. Thickness variations were caused by the influence of SDC powder on castability. The shrinkage of the Ni cathode was equal to 10% and of the Ni-SDC cathode about 3%. This may be due to the addition of the ceramic powder into the system, which was not sintered at the temperature of the firing process.

### 3.3. Porosity Measurements

**Figure 6:**
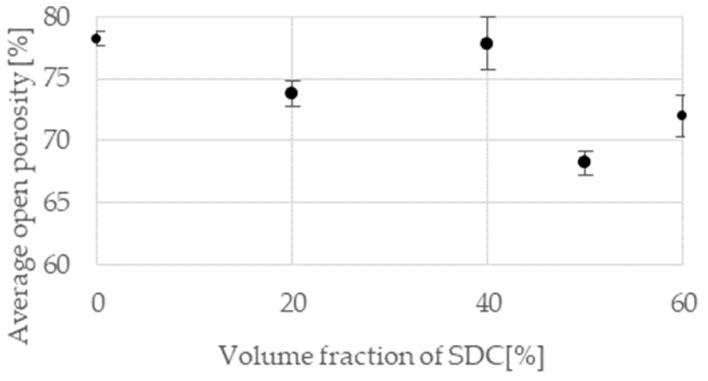
Influence of SDC volume fraction on the average cathode open porosity.

The most important parameter for the cathode in a fuel cell is open porosity. Because of it, the reaction gas can be supplied to the catalyst volume, and this increases the number of reactions taking place on the cathode. As the previous research shows [31], it is not advisable to maximize the porosity but to achieve the optimal porosity using a combination of different pore sizes. The range of cathode open porosity should be between 60% and 80%. The addition of SDC powder does not significantly affect the open porosity obtained (Figure 6). This is probably because the SDC is evenly distributed over the surface of the nickel. SDC powder particles do not form agglomerates that can close open pores or necks leading to them. The optimal porosity level was maintained.

### 3.4. SEM Observation before and after Sintering

**Figure 7:**
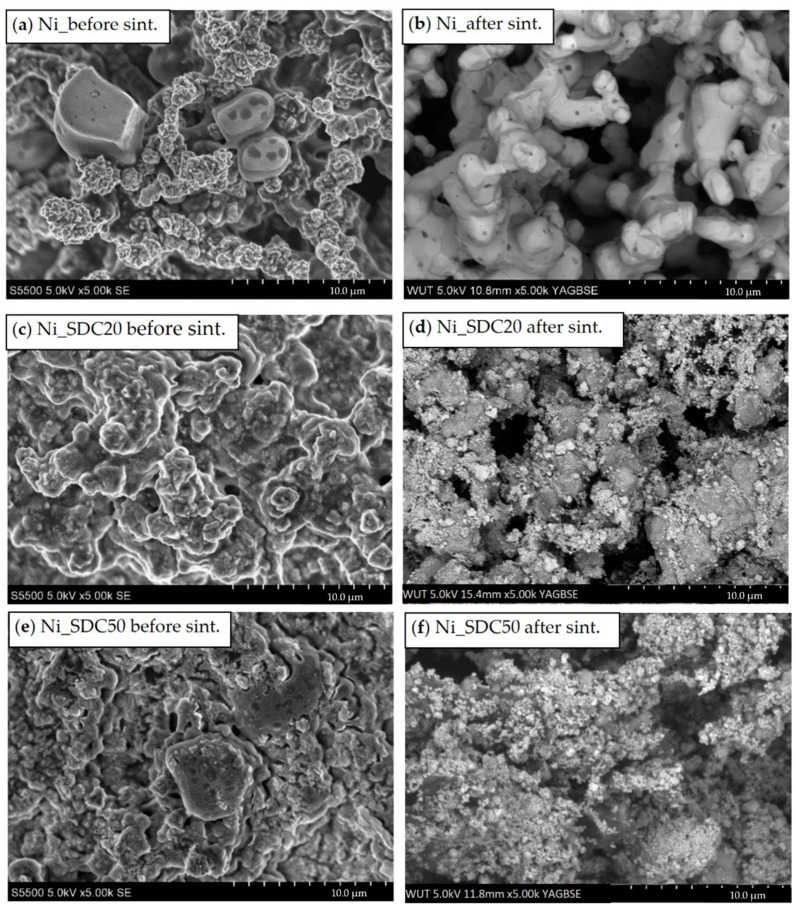
Selected SEM images of the cathode with different chemical compositions before and after sintering (**a**,**b**) cathode with nickel, (**c**,**d**) cathode with nickel and 20% SDC, and (**e**,**f**) cathode with nickel and 50% SDC.

The level of open porosity had not changed, but the morphology of microstructures was different. As seen in SEM images (Figure 7), the polymer base covered the powder particles before sintering. Two types of blowing agents with different morphologies were used, which leave voids after firing. The microstructure of the cathode was very similar to that obtained in other studies performed by T. Wejrzanowski′s team, from which it can be concluded that the pore system is well optimized to improve the oxygen reduction process. The maximum power density obtained for the nickel cathode without foam was 151 mWcm^−2^, and with nickel foam, 231 mWcm^−2^. This indicates that the addition of nickel foam also increases the efficiency of the fuel cell [32]

Optimization of the manufacturing process for the Ni-SDC cathode allowed for the covering of powders by polymers better, which may be due to the longer mixing process and the higher addition of surfactants. Above 40% of SDC, the polymer film was not continuous due to the inferior wettability of the ceramic. After the firing and sintering of the porofors, complex porous microstructures were obtained in the Ni and Ni-SDC cathodes. The sintering temperature of SDC is 1400 °C [33], but Ni starts to sinter earlier, around 600 °C. Due to that, the Ni-SDC cathode microstructure consisted of connected, sintered nickel chains and a fine phase of SDC on the surface. It can be concluded that the specific surface area increases, which is desirable for enhancing catalytic effects.

### 3.5. Specific Surface Area and Porosity

**Figure 8:**
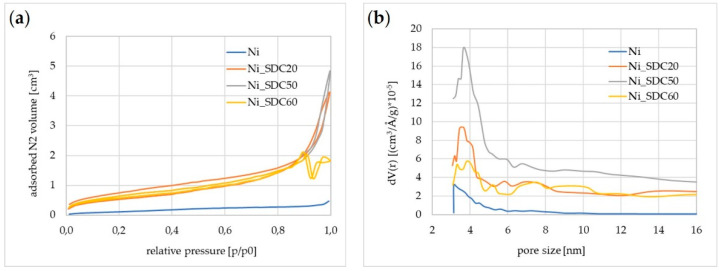
Physical nitrogen sorption analysis results comprising (**a**) obtained sorption isotherms and (**b**) pore size distributions.

The physical nitrogen sorption method was used to study the surface area and porosity of the samples (Figure 8, Table 2). The results show that the cathode Ni with SDC resulted in an expansion of both the specific surface area and porosity. The S_BET_ of Ni_50%SDC was increased by 4 m^2^/g. Since the surface area is generally composed of internal and external area contributions, it was further possible to analyze the S_micropore_ value by applying the statistical thickness method to the data obtained. The results obtained indicate that only for the Ni sample was the S_BET_ divided by an external area and small amounts of micropores having S_micropore_ 0.237 m^2^/g. Covering Ni with SDC completely reduced the micropores to a 0 value. In other words, for all cathodes with SDC, the S_BET_ was related completely to the external surface area. The greatest V_pore_ and D_pore_ values were shown by Ni foam modified with 50% SDC. In summary, the Ni foam modification enabled the evolution of both surface area and microporosity due to the introduction of SDC particles, which should be also beneficial for its activity as a cathode.

### 3.6. XRD Measurement of Cathodes after Sintering in Forming Gas

**Figure 9:**
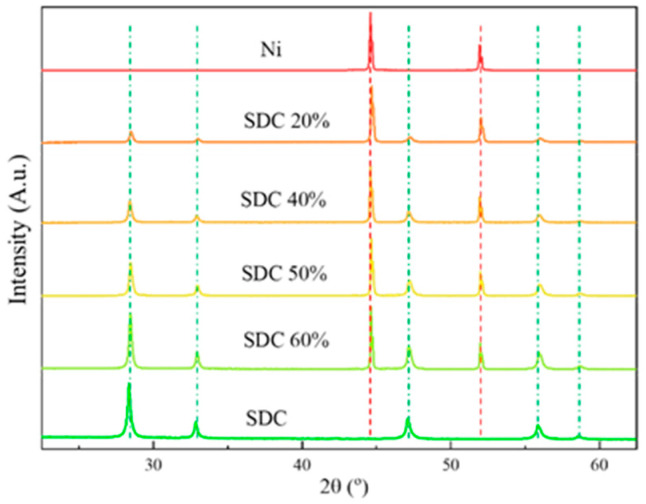
X-ray diffraction patterns of the obtained composite compared with pure Ni and SDC powders. SDC phase is marked with a green dash-and-dot line, Ni phase with a red dashed line.

The phase purity and crystal structure of composite materials were tested using the X-ray diffraction technique. In Figure 9, X-ray diffraction patterns of all the composite materials obtained are presented, juxtaposed with X-ray patterns obtained for pure substrate powders (Ni and SDC). In the case of pure Ni and pure SDC patterns, all reflections can be associated with a Ni cubic structure, the Fm-3m space group marked with red dashed lines, and a SDC cubic structure, the Fm-3m space group marked with green dashed lines, respectively. On the other hand, patterns of composite materials are a mix of both phases, with the intensity dependent on the substrate proportion. It is worth noting that no additional phases are visible in the case of composites, which implies that neither reaction between substrates nor oxidation of Ni occurred during sintering. Similar clear separation of phases were also observed previously when sintered in a reducing atmosphere bulk pellet [34]. Given that during operation, the fuel cell cathode will be exposed to oxidative atmosphere, it is equally important that no additional phases are produced between NiO and SDC. For the composite materials NiO-GDC, no such phases were reported [35,36]. For composites reported in this paper, such studies are in progress and will be the subject of a future paper.

**Figure 10:**
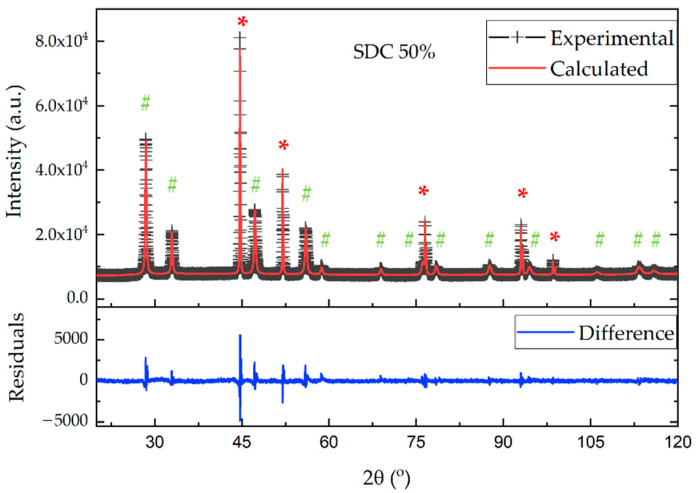
Fitted X-ray diffraction profiles for 50% SDC. Observed (+black symbols), calculated (red line), and difference (blue line, lower) profiles are shown. Reflection positions of Ni and SDC phases are indicated by red “*” and green “#” symbols, respectively.

Microstructural properties of composite materials were additionally verified by a refinement analysis of the X-ray diffraction patterns. As presented in Figure 10, the diffraction profile of the SDC 50% sample can be straightforwardly fitted with a mix of Ni and SDC cubic structure models. Fitted diffraction profiles for the rest of the studied samples, together with refinement parameters, are provided in the supplementary information. Relevant crystal parameters are presented in Table 3. Although the calculated volume fractions of SDC are in good agreement with nominal values, a small increase in Ni content might be due to the presence of Ni foam in the composite. Lattice parameters of the phases present in composite materials deviate slightly from pure powder lattice sizes. This could be related to technological processes (ball milling, sintering in forming gas), which may lead to minor crystal structure deformations. This corresponds to a decrease in the crystallite size of SDC in the composite material.

### 3.7. Mechanical Properties

**Figure 11:**
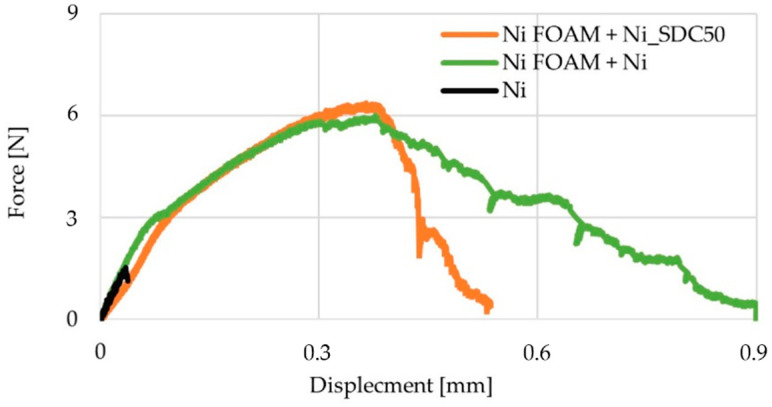
Tensile strength tests for cathodes consisting of: (black line) only Ni layer without Ni foam, (green line) Ni layer with Ni foam, and (orange line) Ni-50% SDC layer with Ni foam.

The mechanical strength of the fuel cell cathode is not a decisive factor in the solution’s applicability since this element is not exposed to high mechanical loads. However, it must be of a minimum strength after sintering to enable it to be handled while the fuel cell is being assembled. Similarly, the cell is compressed in operation and gas pressure is exerted on it. Due to a high porosity and low sintering fraction, the tensile strength of the Ni cathode without Ni foam was deficient (Figure 11).

When the Ni layer was cast on the Ni foam, the tensile strength increased four times, which was shown earlier [27]. The Ni-SDC layer without Ni foam was even more fragile than the nickel layer without a Ni foam support. It was fragile to such an extent that it was impossible to measure it. The introduction of nickel foam increases the strength to a similar level as the layer with only nickel. The use of Ni foam reinforces the Ni-SDC cathode and makes it possible to use it as the cathode in a fuel cell, which would not be possible without the Ni foam.

## 4. Conclusions

The tape casting method was used to manufacture a Ni-SDC cathode with a large surface area. This method allows for the technology to scale to a large extent. Using water as the solvent to prepare the slurry is cheaper and safer for the environment and the operator than an organic solvent. Optimization of the mixing process resulted in a better combination of powders and a polymer base. The open porosity of every cathode was within the optimal range of 60–80% for the molten carbonate fuel cell cathode.

The microstructures of both cathodes differ in morphology, which is influenced by the difference in the sintering temperatures of components. Sintered nickel chains were covered with SDC particles, affecting the specific surface area. Ni powder and SDC did not react with each other during the cathode sintering process. A small deviation of lattice sizes before and after sintering may be related to technological processes (ball milling, sintering in forming gas), which may lead to minor crystal structure deformations. Ni-SDC cathode casting on nickel foam increases its mechanical strength, facilitating the assembly of the fuel cell.

Further studies will be focused on a precise analysis of the sintering process, electrochemical behavior, and composite cathode performance in the fuel cell assembly.

## Figures and Tables

**Table 1:** Characterization of cathode thickness before and after the sintering process.

	Ni	Ni_SDC20	Ni_SDC40	Ni_SDC50	Ni_SDC60
Average cathode thickness after drying (mm)	0.66 ± 0.02	0.67 ± 0.04	0.59 ± 0.01	0.67 ± 0.01	0.62 ± 0.01
Average cathode thickness after sintering (mm)	0.60 ± 0.02	0.65 ± 0.01	0.57 ± 0.01	0.65 ± 0.01	0.59 ± 0.01

**Table 2:** Summary of the results obtained from the physical nitrogen sorption measurements such as the Brunauer–Emmett–Teller surface area (S_BET_), external surface area (S_external_), micropore surface area (S_micropore_), pore volume (V_pore_), and average pore diameter (D_pore_).

Sample	S_BET_ (m^2^/g)	S_external_ (m^2^/g)	S_micropore_ (m^2^/g)	V_pore_ (×10^−3^) (cm^3^/g)	D_pore_ (nm)
Ni	0.463	0.226	0.237	0.72	3.0
Ni_SDC20	2.384	2.384	0.0	6.38	3.4
Ni_SDC50	4.568	4.568	0.0	12.50	5.4
Ni_SDC60	2260	2260	0.0	7.47	3.7

**Table 3:** Relevant crystal structure parameters of various phases in composite materials. Estimated standard deviations are given in parentheses.

	Ce_0.8_Sm_0.2_O_1.9_			Ni
Nominal SDC Fraction (%)	*a* Lattice (Å)	Crystallite Size (nm)	Calculated Volume Fraction (%)	*a* Lattice (Å)
0	-	-	-	3.52555 (1)
20	5.45852 (4)	144 (10)	20.3 (2)	3.52395 (1)
40	5.45853 (4)	84 (3)	37.1 (4)	3.52450 (1)
50	5.45852 (4)	106 (3)	47.0 (5)	3.52425 (1)
60	5.45945 (4)	96 (2)	56.9 (5)	3.52423 (1)
100	5.45816 (3)	225 (12)	-	-

## Data Availability

All data are available from authors after a reasonable request.

## Supplementary Materials

The following supporting information can be downloaded at: https://www.mdpi.com/article/10.3390/ma15144891/s1, Table S1: Crystal and Refinement parameters for all measured composite materials. Estimated standard deviations are given in parentheses; Figure S1: Fitted X-ray diffraction profiles for composite materials. Observed (+ black symbols), calculated (red line), and difference (blue line, lower) profiles are shown. Reflection positions of Ni and SDC phases are indicated by red “*” and green “#” symbols, respectively.
