# Supplementary material for: Fabrication and Characterization of a Composite Ni-SDC Fuel Cell Cathode Reinforced by Ni Foam
Source: Materials (Basel). 2022 Jul 14;15(14):4891. doi: 10.3390/ma15144891 (PMC9319338; doi:10.3390/ma15144891)
Supplement: Supplementary file 1 [file materials-15-04891-s001.zip › materials-1784323-supplementary.pdf]

Fabrication and characterization of composite Ni-SDC fuel cell cathode reinforced by Ni foam

Gabriela Komorowska[1], Tomasz Wejrzanowski[1], Jan Jamroz[2], Agnieszka Jastrzębska[1], Wojciech Wróbel[2], Shu-Yi Tsai[3], Kuan-Zong Fung[3]

[1] Faculty of Materials Science and Engineering, Warsaw University of Technology, Woloska 141, 02-507 Warsaw, Poland
[2] Faculty of Physics, Warsaw University of Technology, Koszykowa 75, 00-662 Warszawa Warsaw, Poland
[3] National Cheng Kung University Department of MSE
*Correspondence: gabriela.komorowska.dokt@pw.edu.pl (G.K.); tomasz.wejrzanowski@pw.edu.pl (T.W.)

Table S1. Crystal and Refinement parameters for all measured composite materials. Estimated standard deviations are given in parentheses.

Sample	Ni 100%	
Chemical formula	Ni	-
Crystal system	Cubic	-
Space group	*Fm-3m*	-
Unit cell dimensions (Å)	*a* = 3.525553(8)	-
Volume (Å^3)	43.821(1)	-
Weight fraction (%)	100	-
Density (g cm^{-3})	8.897	-
R-factors	Phase residuals	
	R_F=0.0167	-
	Total	
	R_{wp}=0.0249	
**Sample**	**Ni 80%, SDC 20%**	
Chemical formula	Ni	$Ce_{0.8}Sm_{0.2}O_{1.9}$
Crystal system	Cubic	Cubic
Space group	*Fm-3m*	*Fm-3m*
Unit cell dimensions (Å)	*a* = 3.52395(1)	*a* = 5.4585(1)
Volume (Å^3)	43.761(1)	162.64(1)
Weight fraction (%)	82.65(7)	17.3(2)

Density (g cm^{-3})	8.909	7.047
R-factors		Phase residuals
	R_F=0.0067	R_F=0.0495
		Total
		R_{wp}=0.0203
Sample	Ni 60%, SDC 40%	
Chemical formula	Ni	$Ce_{0.8}Sm_{0.2}O_{1.9}$
Crystal system	Cubic	Cubic
Space group	*Fm-3m*	*Fm-3m*
Unit cell dimensions (Å)	*a* = 3.52449(1)	*a* = 5.45852(9)
Volume (Å^3)	43.781(1)	162.639(8)
Weight fraction (%)	66.61(15)	33.3(4)
Density (g cm^{-3})	8.905	7.047
R-factors		Phase residuals
	R_F=0.0086	R_F=0.0663
		Total
		R_{wp}=0.0242
Sample	Ni 50%, SDC 50%	
Chemical formula	Ni	$Ce_{0.8}Sm_{0.2}O_{1.9}$
Crystal system	Cubic	Cubic
Space group	*Fm-3m*	*Fm-3m*
Unit cell dimensions (Å)	*a* = 3.52425(1)	*a* = 5.45852(6)
Volume (Å^3)	43.772(1)	162.639(5)
Weight fraction (%)	56.86(16)	43.13(47)
Density (g cm^{-3})	8.907	7.047
R-factors		Phase residuals
	R_F=0.015	R_F=0.0371
		Total
		R_{wp}=0.0200
Sample	Ni 40%, SDC 60%	
Chemical formula	Ni	$Ce_{0.8}Sm_{0.2}O_{1.9}$

Crystal system	Cubic	Cubic
Space group	*Fm-3m*	*Fm-3m*
Unit cell dimensions (Å)	*a* = 3.52423(1)	*a* = 5.45944(4)
Volume (Å^3)	43.772(1)	162.722(4)
Weight fraction (%)	46.13(16)	53.86(47)
Density (g cm^{-3})	8.907	7.044
R-factors	Phase residuals	
	R_F=0.0070	R_F=0.0236
	Total	
	R_{wp}=0.0176	
Sample	SDC 100%	
Chemical formula	-	$Ce_{0.8}Sm_{0.2}O_{1.9}$
Crystal system	-	Cubic
Space group	-	*Fm-3m*
Unit cell dimensions (Å)	-	*a* = 5.45816(17)
Volume (Å^3)	-	162.607(16)
Weight fraction (%)	-	100
Density (g cm^{-3})	-	7.049
R-factors	Phase residuals	
	-	R_F=0.0252
	Total	
	R_{wp}=0.0577	

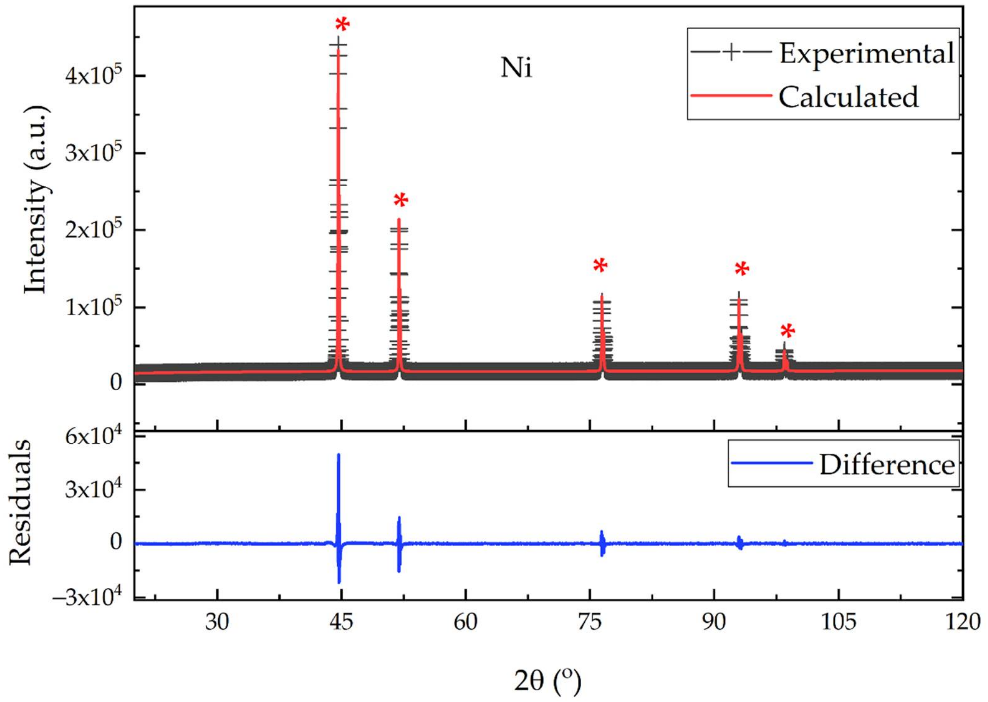
Ni
Experimental
Calculated
Intensity (a.u.)
4x10^5
3x10^5
2x10^5
1x10^5
0
Residuals
6x10^4
3x10^4
0
−3x10^4
Difference
30
45
60
75
90
105
120
2θ (º)

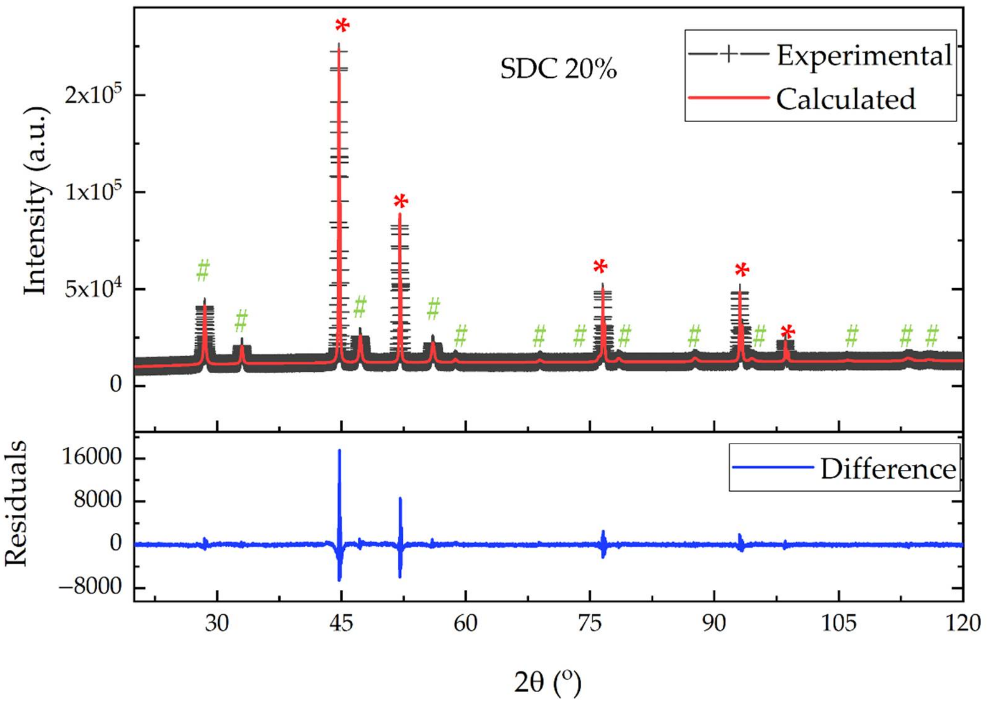
SDC 20%
Experimental
Calculated
Intensity (a.u.)
2x10^5
1x10^5
5x10^4
0
Residuals
16000
8000
0
−8000
Difference
30
45
60
75
90
105
120
2θ (º)

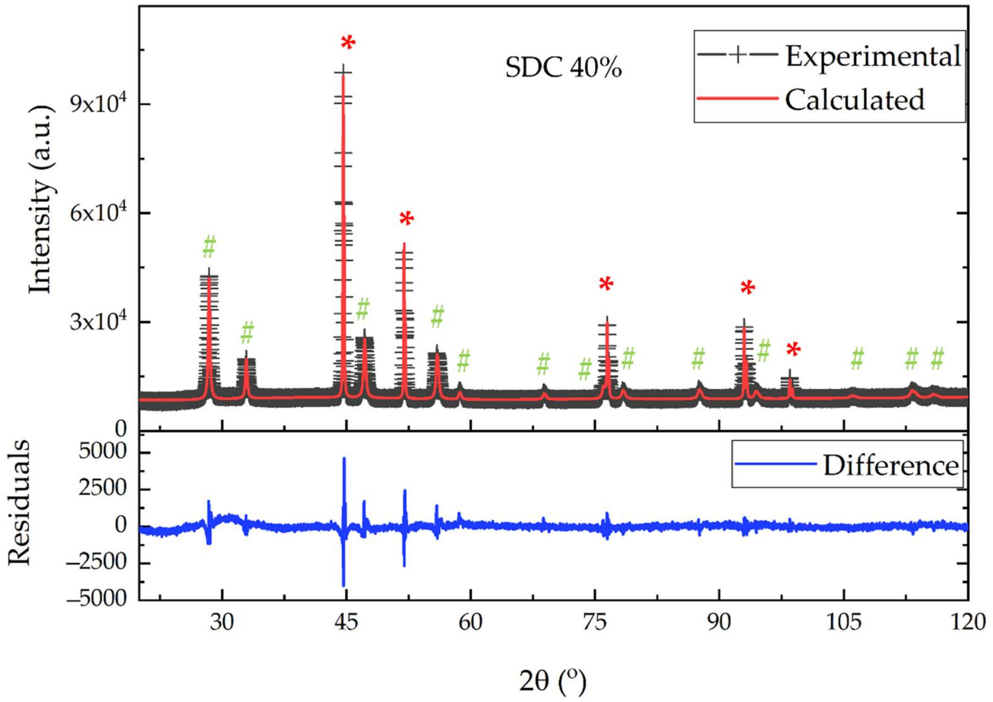

SDC 40%
Experimental
Calculated
Difference
Intensity (a.u.)
Residuals
9x10^4
6x10^4
3x10^4
0
5000
2500
0
−2500
−5000
30
45
60
75
90
105
120
2θ (º)

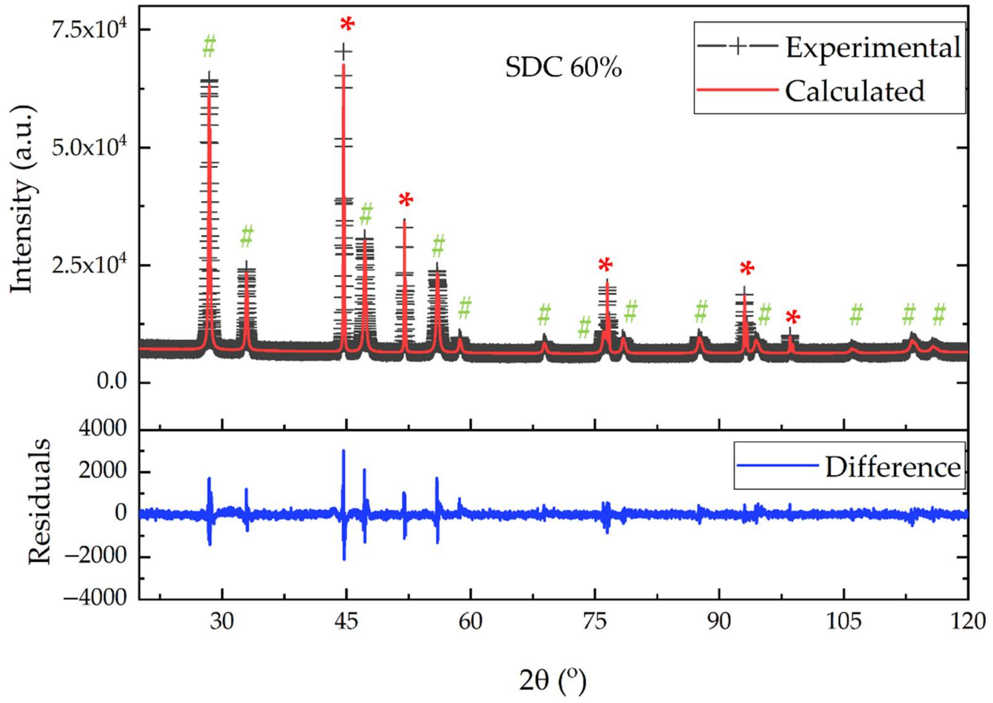

SDC 60%
Experimental
Calculated
Difference
Intensity (a.u.)
Residuals
7.5x10^4
5.0x10^4
2.5x10^4
0.0
4000
2000
0
−2000
−4000
30
45
60
75
90
105
120
2θ (º)

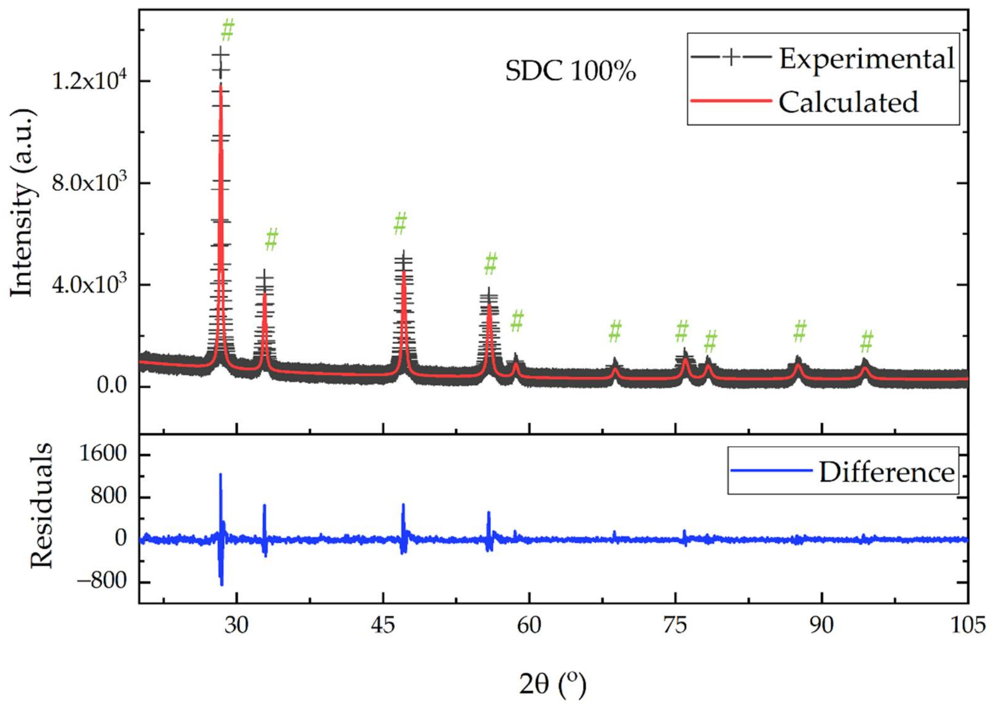

Figure S1. Fitted X-ray diffraction profiles for composite materials. Observed (+ black symbols), calculated (red line), and difference (blue line, lower) profiles are shown. Reflection positions of Ni and SDC phases are indicated by red "*" and green "#" symbols, respectively.
